# Use of Molecular Dynamics for the Refinement of an Electrostatic Model for the *In Silico* Design of a Polymer Antidote for the Anticoagulant Fondaparinux

**DOI:** 10.1155/2013/487387

**Published:** 2013-07-24

**Authors:** Adriana Cajiao, Ezra Kwok, Bhushan Gopaluni, Jayachandran N. Kizhakkedathu

**Affiliations:** ^1^University of British Columbia, Chemical and Biological Engineering, 2360 East Mall, Vancouver, BC, Canada V6T 1Z3; ^2^University of British Columbia, Centre for Blood Research, Department of Pathology and Laboratory Medicine and Department of Chemistry, 2350 Health Sciences Mall, Vancouver, BC, Canada V6T 1Z3

## Abstract

Molecular dynamics (MD) simulations results are herein incorporated into an electrostatic model used to determine the structure of an effective polymer-based antidote to the anticoagulant fondaparinux. *In silico* data for the polymer or its cationic binding groups has not, up to now, been available, and experimental data on the structure of the polymer-fondaparinux complex is extremely limited. Consequently, the task of optimizing the polymer structure is a daunting challenge. MD simulations provided a means to gain microscopic information on the interactions of the binding groups and fondaparinux that would have otherwise been inaccessible. This was used to refine the electrostatic model and improve the quantitative model predictions of binding affinity. Once refined, the model provided guidelines to improve electrostatic forces between candidate polymers and fondaparinux in order to increase association rate constants.

## 1. Introduction

 While anticoagulation therapy is widely used, it has certain undesirable side effects such as the potential to cause life-threatening hemorrhages. Such bleeding complications can be mitigated, in the event of an overdose of anticoagulants, by the administration of antidotes which neutralize the anticoagulants while still avoiding thrombosis [1, 2]. The most commonly used anticoagulants are heparin-derived drugs [3], which include unfractionated heparin (UFH), low molecular weight heparins (LMWHs), and the synthetic pentasaccharide derivatives fondaparinux and idraparinux [4–7]. Because of its predictable dose response, almost complete bioavailability [4, 7], increased half-life [1], and no occurrence of heparin-induced thrombocytopenia [5], fondaparinux is becoming increasingly important in clinical medicine; however, its widespread use is limited by a lack of a specific antidote. Administration of protamine, the antidote for UFH and LMWHs, does not reverse the anticoagulant effect of fondaparinux, and hemodialysis only reduces fondaparinux plasma levels by 20% [1]. Hence, the development of a clinically safe antidote for this anticoagulant has become critical [8].

Currently, only limited experimental work has been reported for the development of an antidote to fondaparinux. It has been shown that heparinase I and the recombinant factor VII (rVIIa) can partially reverse fondaparinux *in vitro*; however, these studies were limited in scope: there is no clinical data for heparinase I, and there is only one volunteer study and one clinical case for rVIIa [1]. More recently, Borgel et al. have experimentally developed antithrombin (AT) variants as potential antidotes for heparin derivatives, including fondaparinux [9, 10]. Although the first of these was shown to neutralize fondaparinux *in vitro* and *in vivo*, its production was severely limited [9]. To overcome this problem, a new chemically modified AT variant has been produced but so far it lacks critical clinical data such as pharmacokinetics, safety, and immunogenicity [10].

The experimental design of a polymeric antidote for fondaparinux is a daunting challenge due to the multitude of structures that need to be synthesized to arrive at the molecule with optimum binding. Given the large number of possible structural configurations for a polymer antidote, a traditional trial-and-error approach to the development of a novel antidote would be a very expensive, labour-intensive, and time-consuming process. Moreover, obtaining experimental data on the interactions between these polymer structures and fondaparinux is also very difficult. Computer simulations therefore provide the only feasible method to screen putative polymer structures that show promise to be effective antidotes for fondaparinux, even though a rigorous experimental validation of these *in silico* predictions is not viable due to the arduousness of polymer synthesis and characterization.

While fondaparinux has been studied *in silico* to some extent [11], the lack of experimental data for the polymer presents a challenge to the application of computational modeling techniques to antidote polymer design. Computer programs for structure-based design strategies require the use of 3D structures, which are typically generated by X-ray crystallography [12]. However, producing an X-ray crystallographic structure of the polymer candidates is difficult because they do not crystallize under normal conditions.

The aim of this work is therefore to use molecular dynamic (MD) simulations to gain a deeper insight—at a microscopic level—into the interactions between fondaparinux and individual polymer's cationic binding groups. This information will guide the selection of favourable binding groups that will promote improved binding between the polymer antidote and fondaparinux. Furthermore, the knowledge gained from these MD simulations will allow for the improvement of the electrostatic model that we have previously reported to characterize the polymer-fondaparinux complex formation but which, due to the lack of interaction data, contained binding simplifications that consequently overpredicted *k*_*a*_ values [13].

In the next sections, the MD simulations and the calculation of the free energy as well as the main equations of the electrostatic model is explained. Then, the selection of the most promising binding groups based on the results obtained from MD simulations and free-energy calculations is discussed. This is followed by a description of the modifications made to the previously published electrostatic model [13] and a discussion of the impact of these changes on the model's predictions.

## 2. Molecular Dynamics Simulations

 All MD simulations in this work were performed using the commercially available software package Materials Studio 5.5 (MS). Each MD simulation system consisted of one individual fondaparinux molecule interacting with one individual cationic binding group surrounded by water molecules. For each MD simulation, the system under study was first prepared, and then its free energies were calculated. The preparation of each model system was performed on an Intel i5 2400 quad-core, 3.1 GHz computer and took approximately 44 h. The MD calculations of the free energies of the prepared systems were then run using 48 2.66 GHz processors from the Bugaboo cluster maintained by WestGrid and Compute/Calcul Canada; these calculations took, on average, 90 h to complete for each system studied.

### 2.1. Binding Group and Fondaparinux 3D System Preparation

**Figure 1:**
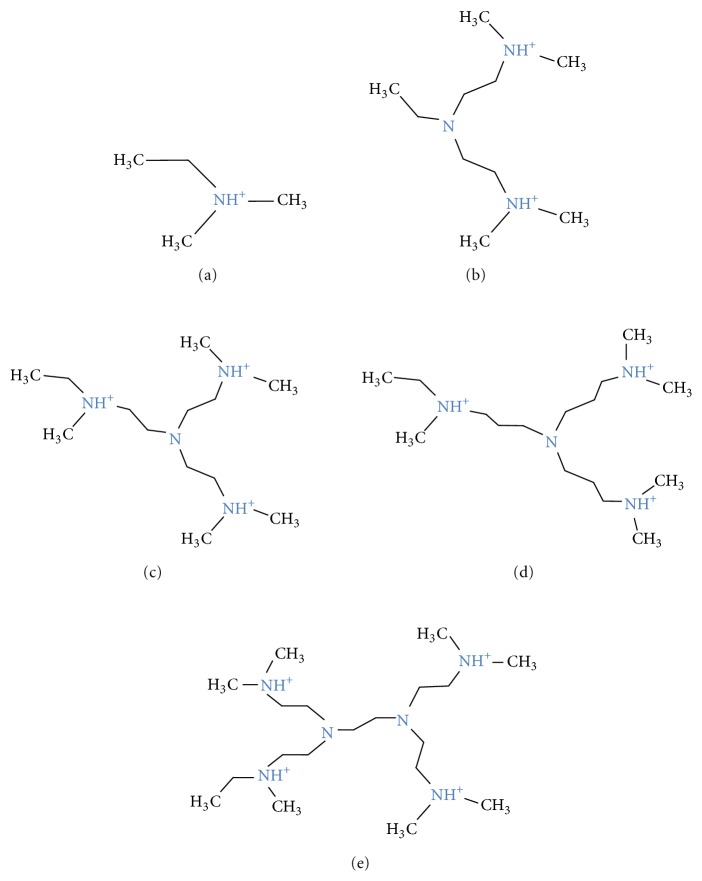
Structures of the various amines used as binding groups: (a) R1-1; (b) R3-1; (c) R4-1; (d) R4-2; and, (e) R6-1. The nitrogen atoms are connected by –CH_2_–CH_2_– linkages in (b), (c), and (e) and by –CH_2_–CH_2_–CH_2_ linkage in (d). The protonation state is for physiological pH of 7.4 and was calculated with ChemAxon pKa Calculator Plugin (Marvin 5.5.5, 2011) [15].

 Because electrostatic interactions drive the binding between fondaparinux and the cationic binding groups, five different binding groups (Figure 1) were chosen in order to determine the effect of valency on complex formation. Based on our previous calculations [13], the R4-1 binding group structure was chosen as the basis for modification. Therefore, the range of theoretically predicted cationic charges was chosen to have values lower and higher than that calculated for R4-1 (i.e., +3). Specifically, binding groups with one, three, four, and six nitrogen, N, atoms connected by –CH_2_–CH_2_– linkages were selected. To observe the structural impact on the binding to fondaparinux, an additional binding group was constructed based on the structure of the binding group R4-1 but consisting of –CH_2_–CH_2_–CH_2_ linkages between the four N atoms.

The initial 3D atomistic structure of fondaparinux was obtained from the DrugBank database [14]. The Na^+^ atoms were deleted from the original 3D atomistic structure in the Visualizer module of MS in order to assign a net charge of −10 to fondaparinux as determined at physiological conditions with the ChemAxon pKa Calculator Plugin (Marvin 5.5.5, 2011) [15]. The partial charges and force field types were assigned with the force field COMPASS using the Discover module. COMPASS is an *ab initio* force field designed for use with a broad range of organic and inorganic molecules and polymers [16] and is optimized for the simulation of condensed phases [17]. As with all current force fields, COMPASS does not properly describe heparin [18, 19], in particular some of the sulfonamide functional groups found within heparin derivatives such as fondaparinux [20]. Therefore, the force field types and charges assigned by COMPASS to the three sulfonamide oxy anions were modified to match those of the undissociated analog and the sulfonylmethoxy oxy anion, respectively. The charges were then adjusted based on a desired net charge of −10 for fondaparinux. Lastly, the structure of fondaparinux was minimized after 575 iterations in the Forcite module of MS using the force field COMPASS. Similarly, the 3D atomistic structures of the different binding groups were sketched using MarvinSketch from ChemAxon (Marvin 5.5.5, 2011) [15]; their protonation state was calculated with ChemAxon pKa Calculator Plugin (Marvin 5.5.5, 2011) [15], and their force field types and partial charges were assigned with the Discover module using the force field COMPASS. The structures were then minimized in the Forcite module of MS using COMPASS.

**Figure 2:**
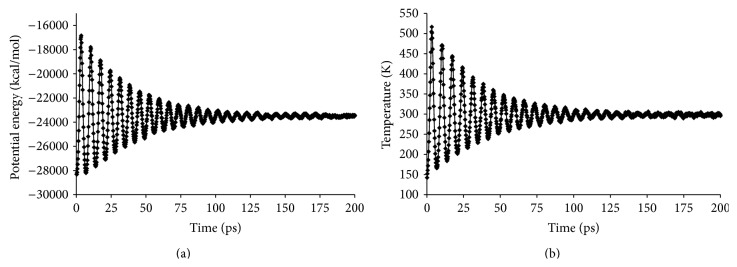
Time evolution of (a) the potential energy (kcal mol^−1^) and (b) the temperature (K) for a model system consisting of 1 R4-1 molecule, 1 fondaparinux molecule, 2,600 water molecules, and 7 Na^+^ atoms.

Each model system was constructed using the Amorphous Cell module of MS and consisted of one deprotonated fondaparinux molecule and one protonated binding group randomly dispersed in 2,600 water molecules. Appropriate amounts of sodium counterions (Na^+^) were added to achieve charge neutrality. A cubic simulation box was constructed with periodic boundary conditions in all directions to avoid surface effects [21, 22], a density of 1 g cm^−3^ since the system consists mainly of water molecules, and a side length of approximately 43 Å. A distance object was created between the center of mass (centroid) of fondaparinux and the centroid of the binding group. Then, a harmonic restraint with a harmonic force constant of 100 kcal mol^−1^ Å^2^ and a harmonic minimum of 21.65 Å was applied to this distance. Energy minimization and MD simulations were performed to equilibrate the system using the force field COMPASS in the Forcite module of MS. The MD simulations were carried out under NVT conditions, with temperature held at 298 K by the Nose-Hoover thermostat [16, 22–24]. The time step was 1 fs, and the simulation time was 200 ps. This simulation time proved to be sufficient to obtain equilibrium conditions, namely, the potential energy and temperature of the system as shown in Figure 2 for the representative system of fondaparinux and an R4-1 molecule. Once the model system was relaxed, the restraint on the centroid-centroid distance was removed from the system.

### 2.2. Free-Energy Calculation

 Using restraint forces, the intermolecular separation of fondaparinux and a binding group was sampled at equal intervals of 0.5 Å [25] from an initial separation of 21.5 Å to a final separation of 1.5 Å. At each interval, a commercially available code distributed by Accelrys was used to solve the following equation [26]:

(1) FR(R′)=−∫Ro′R′〈Kr(R(r)−Ro)〉r,R′′dR′′−2kBTln(R′Ro′)+FR(Ro′),

where *K* ^*r*^ is the harmonic force constant that enhances the restraint of the system to the *R*-coordinate value *R*_*o*_. The reaction coordinate *R* for each system was defined as the distance between the centroid of the fondaparinux molecule and the centroid of the binding group. A harmonic restraint constant of 100 kcal mol^−1^ Å^2^ was applied over this distance. The simulation time at each interval was 60 ps, which consisted of 10 ps of equilibration followed by 50 ps of trajectory generation [27] for a total simulation time of 2.5 ns. The equilibration time was found to be sufficient for properties to equilibrate at each interval. The same NVT conditions, temperature, and time step previously described were used for the free-energy calculations, and each simulation was repeated 5, 4, 7, 8, and 9 times for R1-1, R3-1, R4-1, R4-2, and R6-1, respectively.

## 3. Electrostatic Model for Polymer Antidote and Fondaparinux

 We have previously described an electrostatic model that provided an indication of the binding affinity between fondaparinux and a polymer structure with R4-1 groups [13]. Since the association rate constant, *k*_*a*_, is determined by diffusion and can be increased by favourable electrostatic forces [28–31] whereas the dissociation rate constant, *k*_*d*_, is determined by short-range interactions between the molecules and is independent of long-range electrostatic forces [28, 32], the overall association constant, *K*_*a*_, and thus the affinity of a complex can be increased by optimizing the electrostatic interactions between the molecules [28]. Our model predicts *k*_*a*_ based on these electrostatic interactions and on the following equations, first derived by Schreiber and coworkers [28, 33, 34]:

(2) $$\begin{array}{c} \ln k_{a}=\ln k_{a}^{o}-\frac{\Delta U}{k_{B}T}\left(\frac{1}{1+ka}\right), \end{array}$$

where *k*_*a*_ and *k*_*a*_^*o*^ are the association rate constants in the presence and absence of long-range electrostatic forces, respectively; Δ*U* is the electrostatic energy of interaction; *k*_*B*_ is the Boltzmann constant; *T* is the temperature of the solution; *a* is the minimal distance of approach between the molecules; and *k* is the Debye-Hückel parameter. The Debye-Hückel parameter is defined as [35]

(3) $$\begin{array}{c} k=\sqrt{\frac{2F^{2}I}{\epsilon_{o}\epsilon_{r}RT}}, \end{array}$$

where *F* is the Faraday constant, *I* is the ionic strength of the solution, *ϵ*_*o*_ is the vacuum permittivity, *ϵ*_*r*_ is the dielectric constant of the solution, and *R* is the gas constant. The electrostatic energy of interaction is defined as [28, 32]

(4) $$\begin{array}{c} \Delta U=U_{\text{complex}}-U_{\text{molecule}\;\text{A}}-U_{\text{molecule}\;\text{B}}, \end{array}$$

where *U*, the Debye-Hückel energy of a molecule, can be calculated from

(5) $$\begin{array}{c} U=\frac{1}{2}\sum_{i,j}\frac{q_{i}q_{j}}{4\pi \epsilon_{o}\epsilon_{r}r_{ij}}\frac{e^{-k(r_{ij}-a)}}{1+ka}. \end{array}$$

In this equation, *q*_*i*_ and *q*_*j*_ are the charges of the atoms in the molecules, and *r* is the distance between the charges.

As we have shown previously [13], we extended the empirically proven model to determine *k*_*a*_ for the interactions of fondaparinux and a polymer structure with R4-1 groups. This model considers the polymer and fondaparinux, due to their structures, as a sphere and a rod, respectively. Based on the Smoluchowski limit for the diffusion-controlled association of two uniformly reactive molecules with these geometries, *k*_*a*_^*o*^ can be calculated as [29]

(6) $$\begin{array}{c} k_{a}^{o}=4\pi N_{A}\left(D_{A}+D_{B}\right)R_{x}. \end{array}$$

Here, *N*_*A*_ is the Avogadro constant, *D*_*A*_ and *D*_*B*_ are the diffusion constants of molecules *A* and *B*, respectively, and *R*_*x*_ is the interaction radius. *R*_*x*_ is defined as

(7) $$\begin{array}{c} R_{x}=\frac{l}{\ln\left(2l/w\right)}, \end{array}$$

where *l* and *w* are the major and minor semiaxes of the ellipsoid. The diffusion constants are calculated as

(8) $$\begin{array}{c} D_{A}=\frac{k_{B}T}{6\pi \eta r_{A}^{\prime }},\;\;D_{B}=\frac{k_{B}T}{6\pi \eta r_{B}^{\prime }}, \end{array}$$

where *r*_*A*_′ and *r*_*B*_′ are the hydrodynamic radii and *η* is the viscosity of the solvent.

For modeling purposes, the interaction between the polymer and fondaparinux was assumed to be entirely electrostatic in nature [13]. Also, to simulate the functionality of the polymer, cationic binding groups on the polymer were modeled as randomly distributed on the surface of a sphere with a given radius, *r*_*H*_, and a minimum distance between the binding groups, *r*_min_, was introduced. The purpose of this *r*_min_ was to account for electrostatic repulsions between the binding groups and to avoid placing these binding groups at the same location, which is physically impossible [13]. In order to represent the solution conditions used in experiments, the following parameters were defined: *I* = 150 mM, *T* = 25°C, *η* = 0.90 × 10^−3^ kgs^−1^ m^−1^, and *ϵ*_*r*_ = 80.

To capture the average properties of a large group of individual molecules, each simulation for a given condition consisted of constructing 1,000 unique polymers by randomly attaching the desired number of binding groups over the surface of the polymer. The model equations were then solved for each of the 1,000 polymers, and the average association rate constant was given by the geometric mean of these 1,000 runs. In the case of no polymer-fondaparinux binding, *k*_*a*_ was set to a value of 1.

## 4. Results and Discussions

### 4.1. MD Interaction between Fondaparinux and Candidate Binding Groups

 MD simulations were performed to calculate the potential of mean force (PMF)—the free-energy profile along the reaction coordinate [36] that yields the difference in free energy between the two states of interest [26]. These free-energy differences (~Δ*G*) between the unbound and bound state of the cationic groups are directly related to binding constants [26].

#### 4.1.1. Effect of Cationic Charge per Binding Group on Free Energy

**Figure 3:**
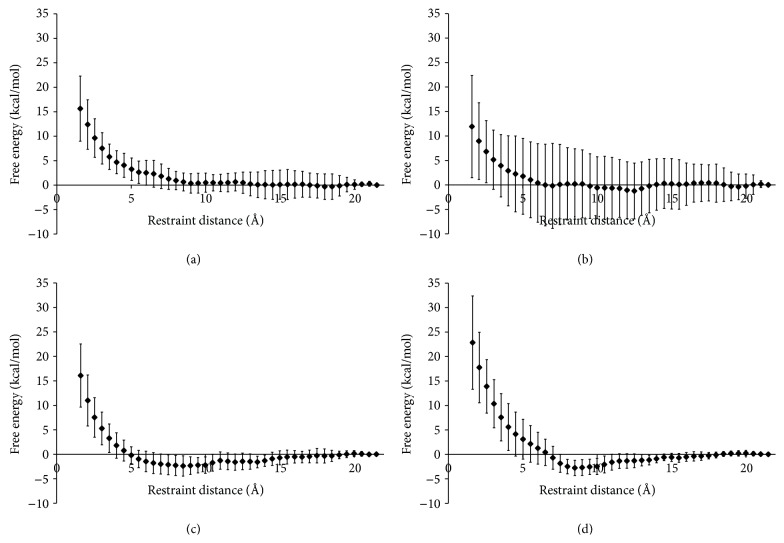
Free-energy profile (PMF) for the interaction of fondaparinux with (a) R1-1, (*n* = 5); (b) R3-1, (*n* = 4); (c) R4-1, (*n* = 7); and (d) R6-1, (*n* = 9). Calculated values are the Helmholtz free energy which approximates the Gibbs free energy for systems in the condensed phase [39, 40]. Simulations were performed using a step size of 0.5 Å and a simulation time of 60 ps at each interval. The error bars represent the 95% confidence intervals for *n* number of replicates.

 In order to investigate the effect of the binding group's charge on fondaparinux binding, MD simulations were run to follow the interaction of fondaparinux and each of the cationic binding groups (Figure 1) in a solution of Na^+^ ions. As shown in their respective free-energy profiles, both of the cationic binding groups R1-1 and R3-1 did not have distinctive energy minima (Figures 3(a) and 3(b)). The lack of free-energy wells indicates weak binding of both R1-1 and R3-1 to fondaparinux. The poor binding of R1-1 was to be expected since it has been shown that the electrostatic interactions of a single protonated amine with a polyanionic molecule are weak and have to compete with salt binding under physiological conditions [37]. R3-1 has an increased charge compared to R1-1 and would therefore be assumed to display improved binding; however, geometry and chemical structure also play a role in complex formation. Therefore, the MD results suggest that R3-1 shows some binding to fondaparinux although it is not sufficient to overcome unfavourable orientations, thus the large degree of variability seen in the PMF for R3-1 compared to R1-1.

Considering the aforementioned weak electrostatic interactions between single protonated amines and polyanionic molecules [37], it is not surprising that the PMF of both R4-1 and R6-1 displayed deeper free-energy wells than the lesser charged R1-1 and R3-1 (Figures 3(c) and 3(d)). In addition, the energy wells of both R4-1 and R6-1 were wide, spanning for 10 Å and 7 Å, respectively, and plateauing at a centroid-centroid distance of approximately 17.5 Å in both cases. This indicates that interactions between R4-1 and R6-1 and fondaparinux were much stronger and therefore felt over a larger distance than those seen in R3-1. Moreover, the variabilities in the PMFs of R4-1 and R6-1 were reduced compared to that of R3-1 which indicates that the electrostatic interactions between fondaparinux and the higher charged binding groups were strong enough to overcome unfavourable orientations.

**Figure 4:**
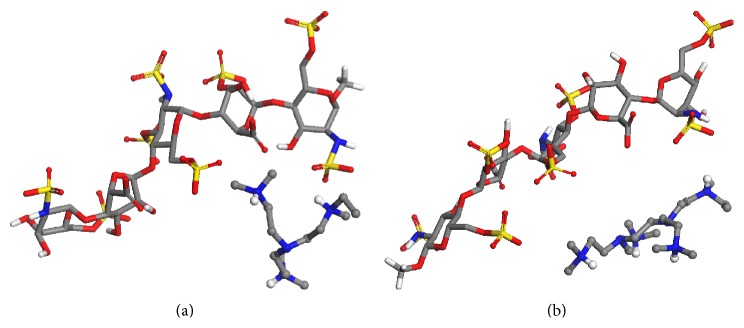
Snapshot of the complexes: (a) R4-1 (ball and stick) and fondaparinux (stick) in a model system consisting of 1 R4-1 molecule, 1 fondaparinux molecule, 2,600 water molecules, and 7 Na^+^ atoms; and (b) R6-1 (ball and stick) and fondaparinux (stick) in a model system consisting of 1 R6-1 molecule, 1 fondaparinux molecule, 2,600 water molecules, and 6 Na^+^ atoms. In both cases the centroid-centroid distance is 8.5 Å. Water molecules and Na^+^ atoms are deleted for clarity.

Since binding affinity can be improved by increasing the cationic charges within the binding group, R6-1 could be expected to show a more favourable energy of interaction with fondaparinux than R4-1. However, both of the binding groups had their local minima at a centroid-centroid distance of 8.5 Å, and both PMF profiles yielded comparable calculated Δ*G* values of −2.394 kcal mol^−1^ for R4-1 and −2.768 kcal mol^−1^ for R6-1. These results suggest that there is not a significant difference between the binding of individual R4-1 and R6-1 binding groups to fondaparinux. However, because of its additional cationic charge it could be hypothesized that when many R6-1 binding groups are working in concert on the surface of the polymer, the small improvement in binding affinity they show compared to R4-1 will be amplified and will provide stronger electrostatic interactions with fondaparinux. This is investigated further below with the electrostatic model. The complexes formed by each of these binding groups and fondaparinux can be observed in Figure 4. 

#### 4.1.2. Effect of Binding Group Structure on Free Energy

 An alternate method of improving binding affinity between binding groups and fondaparinux is to change the spacing of the cationic charges within the binding groups [38]. Shortening the linkage between the N atoms in R4-1 from –CH_2_–CH_2_– to –CH_2_– resulted in the protonation of only one of the amines at pH 7.4 (as calculated with ChemAxon pKa Calculator Plugin (Marvin 5.5.5, 2011) [15]), and therefore, this molecule was deemed unsuitable for further study. Conversely, the theoretical charge of R4-1 was maintained at +3 when the linkage between the N atoms was lengthened from –CH_2_–CH_2_– (3.84 Å) to –CH_2_–CH_2_–CH_2_– (4.97 Å) to form the R4-2 binding group (Figure 1).

**Figure 5:**
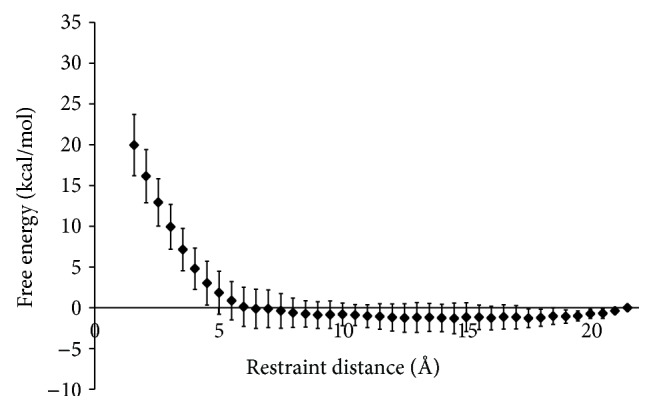
Free-energy profile (PMF) for the interaction of fondaparinux with R4-2 (*n* = 8). Calculated values are the Helmholtz free energy which approximates the Gibbs free energy for systems in the condensed phase [39, 40]. Simulations were performed using a step size of 0.5 Å and a simulation time of 60 ps at each interval. The error bars represent the 95% confidence intervals for *n* number of replicates.

The free-energy profile for R4-2 (Figure 5) displayed a shallow and poorly defined well. Comparing the free-energy profiles of R4-1 (Figure 3(c)) and R4-2 suggests that increasing the spacing of cationic charges for this binding group will not generate an improvement in the binding affinity to fondaparinux. In fact such a structural change is shown to inhibit fondaparinux binding. Therefore, the binding groups R4-1 and R6-1 are considered the most promising binding groups for the effective neutralization of fondaparinux and will be investigated using the electrostatic model.

### 4.2. Refinement of Electrostatic Model to Optimize Polymer Antidote Structure

 The electrostatic model first developed in our previous work [13] is herein refined based on the microscopic information gathered from the MD simulations described above. In the simplified model, the minimal distance of approach, *a*, was set to 6 Å since this value had been shown to give the best fit for experimental data collected for a variety of protein systems [28]. However, the free-energy profiles of the interaction of binding groups R4-1 and R6-1 to fondaparinux (Figures 3(c) and 3(d)) showed that the centroid-centroid distance between a binding group and fondaparinux was 8.5 Å at binding. Since the refined model focused on these binding groups, the value for *a* was changed to 8.5 Å.

The binding criterion used in the simplified model was based on the direct contact of fondaparinux with each of the three closely spaced binding groups that formed a binding site [13]. However, the MD simulation results suggest that during the binding of fondaparinux to either R4-1 or R6-1 the ionic sites of fondaparinux remain at a distance from the protonated N atoms of the binding groups. In addition, it was found that the molecules in a bound complex show significant and constant relative motion to one another. Therefore, the model was refined to incorporate a more realistic binding criterion. A binding site was redefined to be three or more binding groups that are within an area that allows them to come within a prescribed distance (8.5 Å) of a fondaparinux molecule, centered around one of the binding groups. Upon binding, a single charge was then assigned to this binding site (the sum of the charges of fondaparinux and the associated binding groups) at the location of the first binding group that formed the binding site.

In order to account for bridging effects, excluding associated structural modifications that might occur, the model was altered to consider system neutralization rather than fondaparinux neutralization by allowing fondaparinux molecules to bind without the requirement for almost complete neutralization of a bound fondaparinux molecule. The fondaparinux charges that are not involved in binding to the generated polymer are therefore available for binding to another polymer in solution.

The minimum distance between binding groups, *r*_min_, for the refined model was determined as described in our previous publication [13]. The new value of *r*_min_ was found to be 9.16 Å, which is larger than the radius of R4-1 (6.61 Å) and R6-1 (7.10 Å). Since the purpose of introducing an *r*_min_ was to account for electrostatic repulsions and steric interactions between binding groups, this new *r*_min_ is a more realistic constraint than the previously reported value [13], which was smaller than the radius of R4-1.

#### 4.2.1. Effect of Number of Binding Groups on *k*_*a*_ and Number of Molecules of Fondaparinux Bound per Polymer

**Figure 6:**
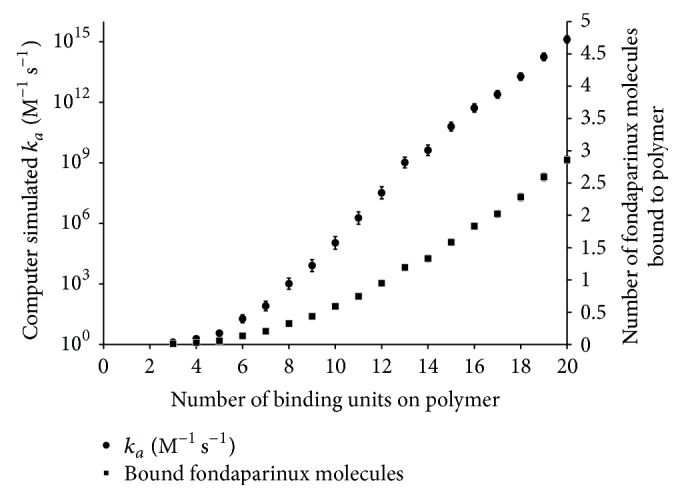
Computer simulated *k*_*a*_ and number of fondaparinux molecules bound per polymer for polymers with 3 to 20 attached R4-1 binding groups. The HBSPCMs had an *r*_*H*_ of 4.0 nm and an *r*_min_ of 9.16 Å. *k*_*a*_ and number of fondaparinux molecules bound to a polymer are the geometric and arithmetic means, respectively, of 1,000 calculated values. The error bars represent the 95% confidence intervals.

 The metric for binding affinity used in this work is *k*_*a*_ since, as previously described, an increase in *k*_*a*_ would increase *K*_*a*_. With the refined model, *k*_*a*_ and the number of fondaparinux bound per polymer increased with the number of attached R4-1 binding groups in similar fashions to what was observed using the simplified model (Figure 6). However, the range for *k*_*a*_ was reduced by 6 orders of magnitude with the refined model compared to the results obtained with the simplified form. These results indicate that although the simplified form of the model predicts overall trends in *k*_*a*_ and thus represents an effective but rough tool for antidote discovery, the refined model with the aid of MD simulation results yields more quantitatively accurate information.

#### 4.2.2. Effect of HBSPCM Size on *k*_*a*_ and Number of Molecules of Fondaparinux Bound per Polymer

**Figure 7:**
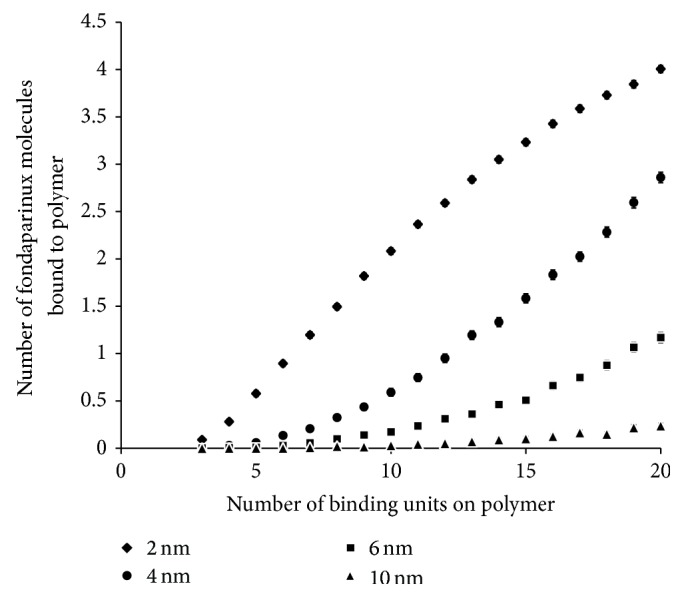
Computer simulated number of fondaparinux molecules bound per polymer for polymers with radii, *r*_*H*_, of 2.0 nm, 4.0 nm, 6.0 nm, and 10.0 nm. All different sized polymers were tested with a number of R4-1 binding groups ranging from 3 to 20 using an *r*_min_ of 9.16 Å. The number of fondaparinux molecules bound to a polymer is the arithmetic mean of 1,000 calculated values. The error bars represent the 95% confidence interval.

**Figure 8:**
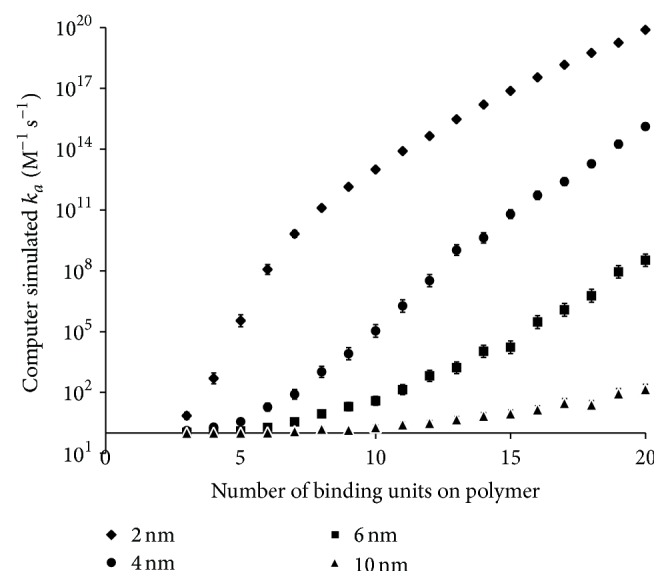
Computer simulated *k*_*a*_ for polymers with radii, *r*_*H*_, of 2.0 nm, 4.0 nm, 6.0 nm, and 10.0 nm. All different sized polymers were tested with a number of R4-1 binding groups ranging from 3 to 20 using an *r*_min_ of 9.16 Å. *k*_*a*_ is the geometric mean of 1,000 calculated values. The error bars represent the 95% confidence interval.

 The effect of polymer size and number of R4-1 binding groups on the number of fondaparinux molecules bound per polymer and *k*_*a*_ was investigated with the improved binding model (Figures 7 and 8). As with the simplified model, it was observed that as the surface area of the polymer core increases, the probability of finding binding groups sufficiently close to each other to form a binding site decreases. The improvements to the model have a greater impact for smaller polymers and as shown in Figure 7, the refined model does not predict a linear increase in the number of fondaparinux molecules bound per polymer with a radius of 2 nm as was the case for the simplified model. Instead, the number of fondaparinux molecules bound per polymer starts to plateau at a high number of binding groups. Similar trends were observed for *k*_*a*_. These results are to be expected as the number of fondaparinux bound to a polymer of a given surface area will eventually reach a maximum due to space limitations.

#### 4.2.3. Impact of Binding Group Effective Charge on *k*_*a*_ and Number of Molecules of Fondaparinux Bound per Polymer

**Figure 9:**
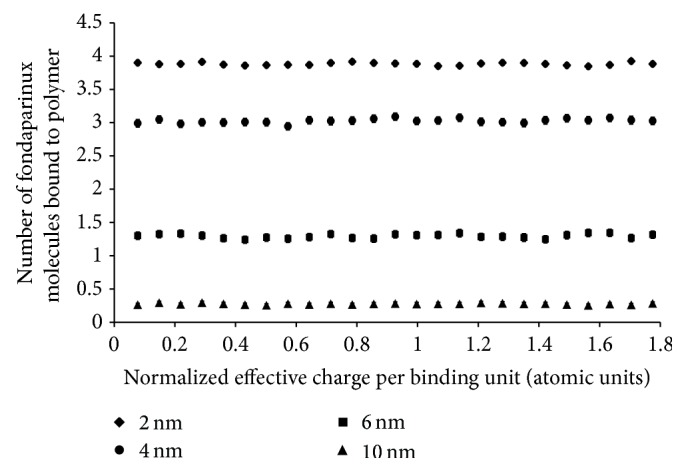
Computer simulated number of fondaparinux molecules bound per polymer for polymers with radii, *r*_*H*_, of 2.0 nm, 4.0 nm, 6.0 nm, and 10.0 nm. All different sized polymers were tested using an *r*_min_ of 9.16 Å with 20 R6-1 binding groups with different effective charges. The number of fondaparinux molecules bound to a polymer is the arithmetic mean of 1,000 calculated values. The error bars represent the 95% confidence interval. The effective charges shown were normalized against the undisclosed effective charge of the R4-1 binding groups.

 Although the effective charge for R4-1 was known *a priori*, it was not known for the R6-1 binding groups. Therefore, the impact of the effective charge of attached R6-1 binding groups on *k*_*a*_ and the number of molecules of fondaparinux bound per polymer was also investigated *in silico*. For the purposes of this investigation, polymers with 20 R6-1 binding groups on their surface were used because charge effects are more easily observed at a high number of binding groups. As seen in Figure 9, the number of fondaparinux molecules bound per polymer was not dependent on the effective charge of the binding groups. This was to be expected as the charge of the binding group does not affect the model's placement of binding groups on the surface of the polymer nor the binding criterion and, thus, will have no impact on an individual fondaparinux molecule finding an appropriate binding site.

**Figure 10:**
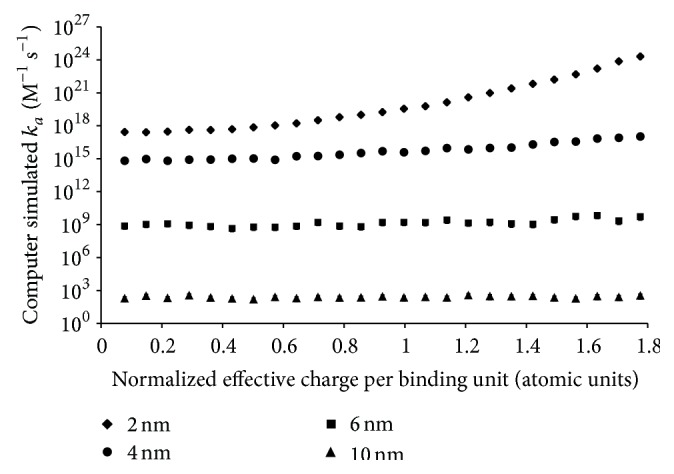
Computer simulated *k*_*a*_ for polymers with radii, *r*_*H*_, of 2.0 nm, 4.0 nm, 6.0 nm, and 10.0 nm. All different sized polymers were tested using an *r*_min_ of 9.16 Å with 20 R6-1 binding groups with different effective charges. *k*_*a*_ is the geometric mean of 1,000 calculated values. The error bars represent the 95% confidence interval. The effective charges shown were normalized against the undisclosed effective charge of the R4-1 binding groups.

Conversely, it was expected that *k*_*a*_ would increase as the effective charge of the binding groups was increased (Figure 10) since *k*_*a*_ is dependent on electrostatic forces and can be increased by increasing the magnitude of these forces [28, 31, 32]. The results show that the increase in *k*_*a*_ with effective charge was most pronounced at an *r*_*H*_ of 2.0 nm and that for polymers with larger radii the effective charge did not have a large impact on *k*_*a*_. This can be explained by the fact that electrostatic energy is inversely proportional to the distance between two charges. As the binding groups become spaced farther apart with increasing polymer radius, the impact of any increase in effective charge becomes dampened.

#### 4.2.4. Effect of Binding Groups on *k*_*a*_ and Number of Molecules of Fondaparinux Bound per Polymer

**Figure 11:**
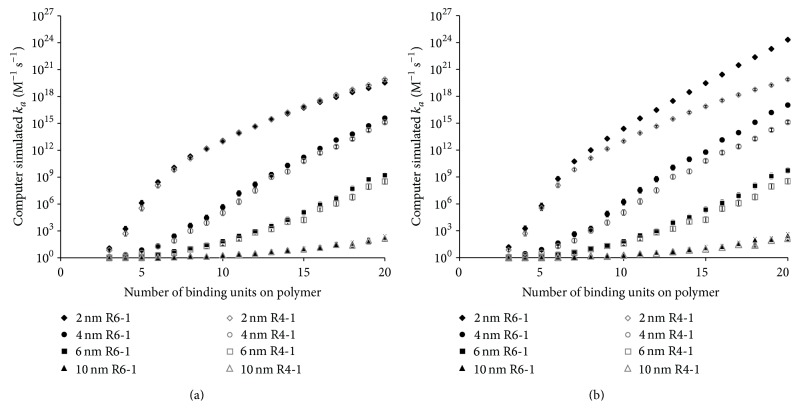
Comparison of computer simulated *k*_*a*_ values for polymers consisting of R4-1 binding groups and R6-1 binding groups with effective charges of (a) a value equivalent to the undisclosed effective charge of the R4-1 binding groups and (b) a value +1 greater than that of R4-1. Polymers of radii, *r*_*H*_, of 2.0 nm, 4.0 nm, 6.0 nm, and 10.0 nm were tested with the number of binding groups ranging from 3 to 10 and using an *r*_min_ of 9.16 Å. *k*_*a*_ is the geometric mean of 1,000 calculated values. The error bars represent the 95% confidence interval.

 The improved model can also be used to determine the effect of replacing R4-1 binding groups with R6-1. As mentioned above, the effective charge of R6-1 is unknown; therefore, model predictions for *k*_*a*_ were made over a range of R6-1 effective charges (Figures 11(a) and 11(b)). When R6-1 has the same effective charge as R4-1 (Figure 11(a)), the model predicts no difference in the calculated values of *k*_*a*_ for R4-1 and R6-1. This indicates that the slightly larger radius of R6-1 does not impact the formation of binding sites to a degree that an effect in the affinity of the polymer to fondaparinux is observed. However, it is likely that the effective charge of the R6-1 binding groups would be higher than that of R4-1 since the slightly larger R6-1 binding groups would most likely experience less charge shielding associated with the polymer's core. The effect of having R6-1 binding groups with higher effective charge is an increase in *k*_*a*_ values, especially for polymers with an *r*_*H*_ of 2.0 nm and 4.0 nm (Figure 11(b)). This result suggests that the binding of the polymer to fondaparinux can be improved by using R6-1 binding groups on small polymer cores. This is consistent with the results of the simplified model, which recommended the synthesis of polymers with low hydrodynamic size in order to increase the charge density of the polymer and, therefore, enhance *k*_*a*_ [13].

## 5. Conclusions

 MD simulations have enabled the refinement of a model that has previously been shown to qualitatively but not quantitatively characterize the binding of fondaparinux to a polymer-based antidote molecule. In particular, the free-energy differences calculated from MD simulations have been used to determine those potential binding groups that will not bind to fondaparinux with high affinity and those that will provide strong binding. The MD simulation results suggest that both R4-1 and R6-1 binding groups show high affinity for fondaparinux.

The refined electrostatic model was also extended to polymers with R6-1 binding groups. It was found that if, as would be expected, the R6-1 binding groups have a higher effective charge than the R4-1 binding groups, improved binding affinity between the polymer and fondaparinux can be achieved. The results of this work therefore indicate that increasing the charge density of the polymer with favourable binding groups will improve complex formation between the polymer and fondaparinux. Therefore, it is recommended to synthesize small polymers containing as many R6-1 binding groups as possible.

The strengths of the model, both in its simplified and refined forms, are twofold: (a) minimal experimental data is required—data that is difficult to obtain for these polymers—and (b) the polymer structural adjustments can be determined *in silico* thus avoiding the costly and time-intensive synthesis of new polymers. In fact, because the synthesis and testing of even one of these polymers is such an arduous task, the model represents the only viable method for comprehensive antidote candidate screening; even without unfeasible, rigorous experimental validation. The research presented in this work is thus a major contribution to the process of finding an antidote for fondaparinux and, therefore, will greatly impact the therapeutic field.
